# Supplementary material for: Brief Exposure to Infants Activates Social and Intergroup Vigilance
Source: Behav Sci (Basel). 2020 Apr 3;10(4):72. doi: 10.3390/bs10040072 (PMC7225933; doi:10.3390/bs10040072)
Supplement: Supplementary file 1 [file behavsci-10-00072-s001.pdf]

Article

Brief Exposure to Infants Activates Social and Intergroup Vigilance

Bobby Cheon [1,2]* and Gianluca Esposito [1,3]

[1] School of Social Sciences (Psychology), Nanyang Technological University, Singapore
[2] Singapore Institute for Clinical Sciences, Agency for Science Technology and Research (A*STAR), Singapore
[3] Department of Psychology and Cognitive Science, University of Trento, Italy

Version April 3, 2020 submitted to Journal Not Specified

Supplementary Information

Study 1 Supplemental Methods/Procedures

0.0.1. Additional participant characteristics.

The 404 participants in the study consisted of 31 African-Americans, 14 Asians, 326 Whites, 20 Latinos, 4 Native-Americans, 6 biracials, and 3 multiracials based on self-report. Within the sample, 218 (53.96%) reported having children. No participants were excluded from analyses.

0.0.2. Face Stimuli.

A total of 40 color pictures of infant and adult human and animal faces were used (10 for each category). Pictures of infant and adult human and animal faces were adjusted for brightness and color-balance using Adobe Photoshop 8.0.1. Specifically, based on brightness histograms, pictures were modified so that the average brightness value of all pixels fell between 125 and 220 cd/m2. After editing, mean brightness of the four picture categories did not differ from one another. Pictures were also corrected using primary color curves by reducing eventual excess of the primary colors. All pictures of humans showed a frontally oriented, neutrally expressive face on a white background; head size was matched across stimuli. Human adult faces consisted of equal numbers of males and females; human infant faces had no cues to distinguish gender. Puppy faces were also frontally oriented. Face stimuli came from public domain databases [1] [2] [3], or were publicly available images taken by a professional photographer (available on public repository) and edited by a Research Assistant (Guilio Gabrieli). To exclude potential influence of attractiveness on brain activity [4] [5], pictures were selected within a larger database (n= 96 with the same characteristics and sources) and rated by 42 adults (19 males, M age= 32.00, SD= 4.25) on a 4-point Likert scale assessing attractiveness. These participants did not report dog phobias. They were recruited by public advertisement and participated in this behavioral experiment only. The stimuli were presented on a laptop (for 3s each) in one of two possible random orders and were interleaved with a of a 4-point scale ranging from unattractive to attractive. Participants verbally responded to each picture, and their responses were recorded by an experimenter out of the participants' view. We then selected 40 stimuli for the experiment that were the same in attractiveness.

0.0.3. Punishment of violent offender measure.

Participants read descriptions of two incidents when a male perpetrator physically assaulted a female victim. Participants were informed that the perpetrator was found guilty participants were asked to choose an appropriate sentence from the following options: i) verbal rebuke, ii)

30 days-probation, iii) 60 days-probation, iv) 90 days-probation, v) 6 month-probation, vi) 1 year-probation, vii) 7 days of detention, viii) 30 days of detention, ix) 90 days of detention, x) 6 month of detention, xi) 12 month of detention, xii) 3 years of detention, xiii) 5 years of detention, xiv) 7 years of detention, xv) 10 years of detention, xvi) 25 years of detention or more, xvii) rights revocation and deportation, xviii) death penalty. The two scenarios were presented as follows: Scenario 1: "On August 24, 2008, David Edmonds was arrested on domestic violence charges for attacking his ex-girlfriend, Stacy Miller. The couple allegedly split three years ago when Stacy began to date a coworker. Friends say that David still harbors romantic feelings for Stacy and was intensely jealous of her new relationship. After calling Stacy repeatedly at work and at home, David appeared at her place of residence and the two began to argue. David apparently lost his temper and struck Stacy in the face, giving her a broken nose. She also suffered a head concussion from the incident. Neighbors heard the altercation and immediately called the police. David was arrested at his home later that night. David was charged with aggravated assault. He had been arrested several times prior to this incident for various other offenses." Scenario 2: "The assault in question took place in a neighborhood of a large city at approximately 10:30 p.m. on June 14, 2008. The perpetrator of the crime was Alonzo Jenkins. The victim, Carol Wilkins, was walking with a female friend when the incident took place. Witnesses say that Alonzo, who was slightly intoxicated at the time, began yelling foul and distasteful comments at Carol. The two ladies decided to ignore Alonzo, which apparently made him angrier, at which point he approached the ladies and began behaving aggressively. When Carol told Alonzo to go away and leave them alone, he became hostile and began to physically assault her. Carol's friend then ran into a nearby bar to ask for help and call the police. When she returned, she found that Carol had been badly injured and suffered a head concussion, some fractured ribs, a broken nose, and required over a dozen stitches. Alonzo had left the scene by the time the police arrived, but was arrested at his home two days later. Alonzo was charged with aggravated assault and battery. This was Alonzo's third criminal offense."

0.1. Study 1 Supplemental Results

0.1.1. Affiliation Ratings.

A one-way analysis of variance (ANOVA) was conducted to examine whether participants differed in the overall composite index of affiliation directed towards the images they viewed across the four face conditions (infants, males, females, puppies). A significant effect of face condition was observed, $F(3, 400) = 74.71$, $p < .001$. Pairwise comparisons revealed that participants endorsed greater overall affiliative responses to puppies ($M = 78.40$, $SD = 19.72$) compared to infants ($M = 66.33$, $SD = 21.90$), females ($M = 49.75$), and males ($M = 45.77$, $SD = 13.65$), p's < .001. Infants also generated significantly greater affiliative responses than females and males, p's < .001. There was no significant difference in affiliative responses towards females and males.

0.1.2. Intergroup Feelings Thermometers.

One-way ANOVAs were conducted to test differences between the face conditions on intergroup bias in global ratings of favorability on the feeling thermometer for one's own ingroup relative to each of the other target groups. Measures of intergroup bias towards each group were computed by subtracting participants' feeling thermometer ratings for each of the target groups (undocumented immigrants, Asian-Americans, White-Americans, African-Americans, Malaysians, Arabs, people from China, people with schizophrenia) from ratings for "people who are the same ethnicity and nationality" as the respondent (the respective ingroup). There was overall no significant effect of face condition on intergroup bias towards any of the target groups, p's > .05, except for towards White-Americans, $F(3, 400) = 2.95$, $p = .03$, such that participants in the puppy faces condition exhibited significantly lower levels of intergroup bias towards this group than participants in the male faces condition, $p = .04$.

0.1.3. Intergroup bias (Semantic Differentials).

One-way ANOVAs were also conducted to test differences between face conditions on intergroup bias towards the diverse target groups relative to one's own ingroup based on semantic differential ratings. Measures of intergroup bias were computed by subtracting the participants' semantic differential ratings for each target group from semantic differential ratings from one's own ingroup (same ethnicity/nationality). Unlike the feeling thermometer measure, higher scores on the semantic differential ratings reflected greater perceptions of negative and threatening characteristics; thus, higher values on this difference score reflect greater levels of biases against the target group compared to the ingroup. There was no significant effect of face condition on intergroup bias toward any target group relative to one's ingroup, p's > .05.

0.1.4. Punishment of Violent Offenders.

One-way ANOVAs revealed that there was no significant effect of face condition on severity of punishment selected for the perpetrator on either scenario, p's > .05.

0.1.5. Parental Bonding Instrument and Adult Attachment Questionnaire.

One-way ANOVAs revealed there were no significant differences between participants across the face conditions on any subscales of the Parental Bonding Instrument and Adult Attachment Questionnaire, p's > .05.

0.2. Study 2 Supplemental Methods/Procedures

0.2.1. Participants.

The study involved participants study recruited through a database of volunteers available through the University of Trento website and by posting the announcement on social media. Three hundred sixty-six people started the survey but only 253 participants who finished it were included in the final sample. Six participants declared to have children; the others were non-parents. Within the sample, 242 participants were born in Italy, 10 in other Countries (Albania = 3, Russia = 1, Romania = 2, Swiss = 1, South America = 3) and 1 did not answer. However all participants claimed to be mainly surrounded by a western-culture environment, and participants not born in Italy claimed to have been residing in Italy for at least 9 years.

0.2.2. Face stimuli.

Pictures of the neutral faces were presented in black and white with the size of 628p x 648p. Inscribed in the rectangle a circle (circumference = 69cm, diameter = 22cm) was inserted. The space between the inscribed circle and the external rectangle was filled with plain gray color in order for participants to see the most important features of the face (eyes, nose and mouth) excluding other incidental features, such as hair or face's contour. All pictures were completely desaturated. Face stimuli came from public domain databases [1] [2] [3], or were publicly available images taken by a professional photographer (available on public repository) and edited by a Research Assistant (Guilio Gabrieli). Given the similarity between Caucasian and Arabic faces, a separate pilot study was run prior to Study 2 in order to select the most recognizably Arab faces. In the pilot study 36 images were presented to 75 participants born in Italy and living in an Italian environment. After each face was presented, participants were asked the following open question: "What is this infant's nationality?". The 8 infant faces which were mainly recognized as "Arab" were included as stimuli in the present study.

0.2.3. Manipulation Check.

After the all the questionnaires, participants were asked to recall and identify the ethnicity of the infant faces that they were shown at the beginning of the experiment (without being exposed to the faces again). Participants answered 6 multiple choice questions about their memories of the previously viewed images. Participants were asked to decide on how many infant faces, from 0 to 8, they saw from a particular ethnic group. The addressed ethnic groups were: Caucasian, African, Chinese, Arabic, Hispanic, and Indian. The presentation of these six questions was randomized across subjects. Participants who had not previously seen any infant face, such as in the control group where they saw only puppies, skipped these questions.

0.3. Study 2 Supplemental Results

0.3.1. Affiliation Ratings.

A one-way ANOVA revealed that participants in the different face rating conditions differed in ratings on the overall composite index of affiliative responses to the faces, $F(3, 249) = 5.85$, $p = .001$. As in Study 1, affiliation ratings were higher for puppies ($M = 66.99$, $SD = 19.48$) than White infants ($M = 57.35$, $SD = 13.54$, $p = .002$), Asian infants ($M = 57.93$, $SD = 18.15$, $p = .003$), or Arab infants ($M = 55.34$, $SD = 15.51$, $p < .001$). There were no significant differences in affiliation ratings between the White, Asian, and Arab infant conditions, p's > .05.

0.3.2. Intergroup Feeling Thermometers.

Global intergroup biases based on feeling thermometer ratings relative to the ingroup were computed in the same way as in Study 1. A series of one-way ANOVAs revealed that there were no significant differences in intergroup biases in feelings towards the ingroup relative to any of the target groups, p's > .05.

0.3.3. Punishment of Violent Offenders.

One-way ANOVAs revealed that there was no significant effect of face condition on severity of punishment selected for the perpetrator on either scenario, p's > .05.

0.3.4. Worldviews and ideologies.

One-way ANOVAs demonstrated that there were no significant effects of face condition on belief in a dangerous world, competitive jungle beliefs, or right-wing authoritarianism, p's > .05.

0.3.5. Parental Bonding Instrument and Adult Attachment Questionnaire.

One-way ANOVAs revealed there were no significant differences between participants across the face conditions on any subscales of the Parental Bonding Instrument and Adult Attachment Questionnaire, p's > .05.

0.3.6. Interaction between Affiliative Motives and Face Condition.

Unlike Study 1, we observed overall no significant interactions on intergroup bias or social attitude measures, p's > .10. A single exception was a significant interaction between face condition and affiliative motivations on competitive jungle beliefs (model: $F(3, 249) = 6.77$, $R2 = .08$, $p < .001$; interaction: $b = -.017$, $p = .03$), such that while participants who viewed White infant faces exhibited no significant relationship between affiliative motivations and competitive jungle beliefs, $b = .004$, $p = .60$, those who viewed the other faces (outgroup infants and puppies) exhibited a significant negative relationship between affiliative motivations and competitive jungle beliefs, $b = -.01$, $p < .001$.

0.4. Supplemental Discussion

There were some measures that we did not observe effects of the facial condition on across the two studies. The first was severity of punishments selected for a violent perpetrator. Our results on other measures (e.g., BDW, perceived intergroup threat, conservatism) suggest that vigilance associated with exposure to ingroup infant faces may be more likely to manifest by disambiguating the social environment and potential outsiders as actually serving as a source of threat. Although one may expect exposure to infants to promote selection of more severe punishments, the perpetrators described in this measure reflect an unambiguous and very salient threat, such that participants may have been willing to select equally severe punishments regardless of subtle changes in defensive or vigilant motives. Furthermore, the perpetrators were also presented as captured, charged, and found guilty of their crimes, which may obviate the need or urgency to act upon increased vigilance and defensive motivations associated with exposure to infants. Across the two studies, we also did not observe an effect of face condition on intergroup biases manifesting on the feeling thermometer measure. Despite increased intergroup bias based on semantic differential ratings against groups typically considered as being threatening (undocumented immigrants and Arabs) in Study 2, this heightened intergroup bias towards these groups did not emerge on intergroup biases measured with the feeling thermometer. This may be due to the feeling thermometer measuring intergroup bias as a general and global impression of favorability towards the outgroup, whereas the semantic differentials measured bias in a manner that was much more specifically linked to traits rooted in perceived threat (i.e., nice-awful, safe-dangerous, moral-immoral, honest-dishonest). Given that exposure to infants is hypothesized to increase vigilance towards potential sources of threats, the semantic differentials may have been a much more sensitive measure of the quality of intergroup bias elicited by face image manipulation rather compared to a more global and general measure of intergroup bias.

References

1. Cvl face database. *Computer vision lab., faculty of computer and information science, University of Ljubljana, Slovenia.*, available at http://www.lrv.fri.uni-lj. si/facedb.html (2005).
2. Solina, F., Peer, P., Batagelj, B., Juvan, S., & Kovač, J. Colour-based face detection in the '15 seconds of fame' art installation.. *Proceedings of Mirage INRIA*,, 38-47,(2003).
3. Van Duuren, M., Kendell-Scott, L., & Stark, N. Early aesthetic choices: Infant preferences for attractive premature infant faces.. *International Journal of Behavioral Development***27**,212-219, (2003).
4. Parsons, C. E., Young, K. S., Kumari, N., Stein, A., & Kringelback, M. L. The motivational salience of infant faces is similar for men and women. *PloS one***6**,e20632,(2011).
5. Yamamoto, R., Ariely, D., Chi, W., Langleben, D. D. & Elman, I. Gender differences in the motivational processing of babies are determined by their facial attractiveness. *PLoS One***4**,e6042, (2009).

Table 1. Means and standard deviations (in parentheses) across the four face stimuli conditions (females, males, infants, and puppies) in Study 1. Asterisk notation (*) indicates that the mean for a given facial stimuli condition (females, males, puppies) differs significantly from the infant facial stimuli condition ($p < .05$).

	**Infants**	**Males**	**Females**	**Puppies**
Punish-White	10.55 (2.39)	10.06 (2.65)	10.47 (2.91)	10.23 (2.52)
Punish-Black	12.37 (2.07)	12.01 (2.46)	11.98 (2.60)	11.91 (2.05)
White-Thermometer Bias	2.53 (7.38)	3.23 (16.32)	2.55 (12.37)	-1.37 (11.80)*
Black-Thermometer Bias	11.74 (20.58)	17.13 (24.36)	14.22 (25.24)	8.90 (22.21)*
Asian-Thermometer Bias	8.20 (19.06)	11.33 (17.01)	8.61 (21.18)	5.68 (21.10)
Immigrant-Thermometer Bias	32.39 (32.93)	34.59 (29.12)	30.34 (34.10)	24.80 (32.88)
Malay-Thermometer Bias	15.83 (23.24)	21.96 (21.90)	20.00 (25.58)	15.79 (23.40)
Arab-Thermometer Bias	29.45 (30.78)	35.44 (32.16)	30.94 (34.31)	27.65 (33.82)
Schizophrenia-Thermometer Bias	29.79 (28.77)	36.28 (27.49)	28.03 (30.35)	23.04 (31.20)
China-Thermometer Bias	14.67 (22.42)	17.24 (20.74)	16.71 (26.39)	11.07 (23.06)
White-Intergroup Bias	.07 (.35)	.12 (.67)	.16 (.58)	.14 (.53)
Black-Intergroup Bias	.54 (1.17)	.62 (1.28)	.53 (1.27)	.47 (1.11)
Asian-Intergroup Bias	.13 (.85)	.25 (.83)	.20 (.80)	.19 (.76)
Immigrant-Intergroup Bias	1.39 (1.77)	1.40 (1.70)	1.30 (1.71)	1.25 (1.74)
Malay-Intergroup Bias	.51 (1.07)	.62 (1.12)	.68 (1.08)	.54 (1.05)
Arab-Intergroup Bias	1.39 (1.73)	1.49 (1.96)	1.54 (1.92)	1.44 (1.92)
Schizophrenia-Intergroup Bias	1.24 (1.56)	1.39 (1.70)	1.24 (1.49)	1.22 (1.65)
China-Intergroup Bias	.50 (1.13)	.52 (1.10)	.45 (1.13)	.59 (1.08)
PBI-Maternal Care	16.43 (5.86)	17.30 (6.45)	17.19 (6.27)	16.38 (5.64)
PBI-Maternal Overprotection	25.77 (6.90)	25.68 (6.75)	26.36 (6.40)	25.60 (6.80)
PBI-Paternal Care	19.95 (7.79)	19.45 (7.57)	21.19 (8.69)	20.30 (7.23)
PBI-Paternal Overprotection	28.94 (5.97)	28.32 (6.68)	28.78 (6.88)	27.88 (6.71)
ASQ-Confidence	33.31 (7.03)	32.92 (7.24)	32.79 (7.88)	34.21 (7.16)
ASQ-Fear of Intimacy	35.30 (8.92)	36.06 (10.54)	36.69 (10.62)	35.90 (9.17)
ASQ-Relationship as Secondary	18.79 (6.36)	20.67 (6.80)*	18.73 (6.57)	19.18 (6.22)
ASQ-Need for Approval	20.02 (6.91)	20.77 (6.53)	20.25 (7.21)	21.30 (6.64)
ASQ-Preoccupation	26.20 (3.58)	27.06 (4.07)	24.96 (8.55)	26.98 (3.86)

Table 2. Means and standard deviations (in parentheses) across the four face stimuli conditions (Whites, Arabs, Asians, and puppies) in Study 2. Asterisk notation (*) indicates that the mean for a given facial stimuli condition (Arabs, Asians, puppies) differs significantly from the White infant facial stimuli condition (p < .05).

	**Whites**	**Arabs**	**Asians**	**Puppies**
Punish-White	11.59 (2.75)	11.05 (3.19)	11.05 (3.33)	10.87 (3.71)
Punish-Black	14.54 (2.26)	13.98 (2.68)	13.83 (2.33)	13.72 (2.95)
White-Thermometer Bias	10.84 (22.17)	8.73 (16.01)	12.55 (17.69)	9.55 (17.40)
Black-Thermometer Bias	13.68 (20.45)	11.80 (23.45)	14.84 (22.86)	13.05 (19.73)
Asian-Thermometer Bias	17.62 (21.14)	15.44 (23.19)	13.36 (16.10)	15.78 (19.58)
Immigrant-Thermometer Bias	28.90 (31.01)	23.27 (30.37)	24.45 (25.59)	22.80 (26.09)
Malay-Thermometer Bias	18.29 (20.90)	16.52 (20.57)	17.14 (19.50)	15.37 (20.48)
Arab-Thermometer Bias	30.30 (28.57)	24.97 (28.77)	25.48 (23.11)	21.30 (20.88)
Schizophrenia-Thermometer Bias	19.44 (29.07)	23.55 (24.02)	22.53 (24.59)	15.27 (21.66)
China-Thermometer Bias	17.78 (28.82)	20.09 (25.02)	20.19 (18.53)	18.10 (21.92)
PBI-Maternal Care	17.70 (2.11)	17.74 (3.39)	18.30 (2.43)	18.13 (2.22)
PBI-Maternal Overprotection	19.21 (3.79)	19.35 (4.47)	19.39 (3.65)	19.33 (3.92)
PBI-Paternal Care	19.16 (3.34)	18.27 (3.93)	18.83 (3.46)	18.83 (3.10)
PBI-Paternal Overprotection	19.73 (4.98)	20.15 (5.04)	19.86 (4.31)	19.85 (3.72)
ASQ-Confidence	28.79 (6.21)	29.35 (5.58)	29.31 (5.23)	28.98 (5.96)
ASQ-Fear of Intimacy	35.14 (7.74)	36.55 (7.29)	35.53 (6.90)	36.00 (6.94)
ASQ-Relationship as Secondary	16.44 (5.02)	16.67 (5.02)	18.14 (4.79)	18.33 (5.56)
ASQ-Need for Approval	23.18 (5.34)	23.68 (6.44)	23.44 (5.78)	21.05 (6.16)
ASQ-Preoccupation	30.84 (6.08)	30.44 (6.08)	29.56 (6.77)	28.60 (5.94)

© 2020 by the author. Submitted to *Journal Not Specified* for possible open access publication under the terms and conditions of the Creative Commons Attribution (CC BY) license (http://creativecommons.org/licenses/by/4.0/).
